# Response to Dr. Nicholas A. Flavahan’s and Dr. Ali H. Eid’s letters regarding our recent publication in *Cell Genomics*

**DOI:** 10.1016/j.xgen.2025.100885

**Published:** 2025-05-14

**Authors:** Anniina Tervi, Markus Ramste, Hanna M. Ollila

**Affiliations:** 1 Institute for Molecular Medicine Finland, FIMM, Helsinki Institute of Life Science - HiLIFE, University of Helsinki, Helsinki, Finland; 2 Division of Cardiovascular Medicine, Stanford University School of Medicine, Stanford, CA 94305, USA; 3 Faculty of Medicine and Health Technology, BioMediTech, Tampere University, Tampere, Finland; 4 Department of Internal Medicine, University of Helsinki, Helsinki, Finland; 5 Center for Genomic Medicine, Massachusetts General Hospital, Boston, MA, USA; 6 Broad Institute of MIT and Harvard, Cambridge, MA, USA; 7 Anesthesia, Critical Care, and Pain Medicine, Massachusetts General Hospital and Harvard Medical School, Boston, MA, USA

## Main text

Thank you to Dr. Flavahan and Dr. Eid for their thoughtful responses to our recent paper, “Genetic and functional analysis of Raynaud’s syndrome implicates loci in vasculature and immunity,” published in *Cell Genomics*. We appreciate the opportunity to engage in scientific discourse and discuss the methodology and conclusions presented in our study.

### Genetic risk factor and role of *ADRA2A*

We fully acknowledge and appreciate Dr Flavahan’s and Eid’s expertise regarding the complex roles of α2 adrenoceptors (ARs) in cold-induced vasoconstriction. In our study, *ADRA2A* emerged as the major genetic risk factor for Raynaud’s phenomenon (RPh), with consistent association observed across four independent cohorts,^1^ and agrees with earlier genetic studies on RPh.^2^ Genome-wide association studies (GWASs) can identify novel and unexpected biological mechanisms. The robust genetic signal at the *ADRA2A* locus highlights the significant contribution of *ADRA2A* to the pathophysiology of RPh.

Our findings suggest that variations in α2A-AR expression may heighten vasoconstrictive responses, particularly under cold conditions, which are characteristic of Raynaud’s attacks. While we recognize the earlier research of α2C-ARs in cold-induced vasoconstriction,^3^^,^^4^^,^^5^^,^^6^ our genetic analysis focused on the impact of *ADRA2A*. These findings do not minimize the role of α2C-AR but instead suggest an independent role of α2A-AR in vascular contraction and temperature-dependent control of vascular tone. Instead, our results indicate that the genetic risk associated with *ADRA2A* likely modulates the disease phenotype and shows an additional and independent role of *ADRA2A* in RPh.

Additionally, we want to stress that the primary focus of our study was to explore how GWAS-derived associations may mediate the risk for RPh.

### Functional assay to assess adrenergic vasoconstriction

We appreciate Dr. Flavahan’s and Dr. Eid’s thoughtful feedback about the functional assay we used to study adrenergic vasoconstriction in smooth muscle cells (SMCs). It is correct to point out that the cultured SMCs used in our experiments may not reflect the contractile behavior of differentiated native cells. This is, however, a general limitation seen in cell culture models. Moreover, we used significant resources trying to obtain earlier generated cutaneous microvascular SMC lines that had been used for studying microvascular contraction, but we were unable to obtain them. Therefore, we selected pulmonary microvascular SMCs after screening dozens of different SMC lines, as the pulmonary SMCs had expression profiles and cellular function similar to those of α2A-AR and α2C-AR^1^ and microvascular cutaneous SMCs, respectively. We believe that our approach provides valuable insights into the molecular pathways involved in RPh.

Moreover, our primary goal was to explore the role of *ADRA2A* in RPh. Although the assay may not directly replicate *in vivo* responses, it does reveal potential genetic and cellular mechanisms. The changes in collagen remodeling activity upon modulation of α2A-AR expression were particularly interesting and may suggest broader implications for tissue fibrosis and smooth muscle function in the context of RPh. In response to Dr. Eid’s comments, we would like to add that the cells used in the study were derived from female donors and cultured in growth-supplemented smooth muscle basal medium (including FBS) (Lonza # CC-3182), and the serum was starved for 24 h before the contraction assay. We agree that complementary assays, such as those directly assessing immediate contractile responses in cutaneous blood vessels, would strengthen these findings and might provide an exciting future avenue, particularly if we can generate or obtain human cutaneous SMCs. This approach could serve as an additional strategy to investigate cellular contractility and determine specifically whether α2A-ARs elicit a similar response to contraction upon cold exposure in human cutaneous SMCs as observed in our study^1^—particularly, since it has already been shown that the location of these receptors does not change upon cold exposure in rat SMCs or human embryonic kidney cells (HEKs)^3^^,^^4^; nor do they react as α2C-ARs do to induced cell stress in human cutaneous SMCs.^5^ It would also be of great importance to study the contractile profile in patient cells from RPh human cutaneous SMCs compared to healthy controls to elucidate the biological mechanisms underlying RPh.

### Adrenergic vasoconstriction mechanism in RPh

As noted, we were exploring whether genetic variation in *ADRA2A* might modulate the sensitivity of vascular SMCs to adrenergic stimulation, which could then lead to altered vasoconstriction. While it is well established that α2A-ARs mediate vasoconstriction, our study identified specific genetic variants that may influence receptor expression and function in a disease context. Although we agree that our approach was not designed to fully address immediate contractile activity and we plan to examine this in subsequent studies, we believe that the genetic insights provided, especially the major role of *ADRA2A*, are valuable for understanding how adrenergic pathways may be altered in RPh. We would also like to point out that a similar SMC contraction assay has been previously used by several research groups to assess SMC contractility.^7^^,^^8^ Additionally, regarding the cold temperature conditions, we considered multiple temperature options. However, earlier studies^8^ have suggested 28°C in the cell incubator as an appropriate condition to mimic the effects of cold exposure in skin tissue.

### Potential role of *IRX1* and other loci in RPh

Dr. Flavahan highlighted the association of the *IRX1* locus with RPh and raised interesting points regarding its potential role in regulating non-shivering thermogenesis and adipose tissue function. We share his interest in this intriguing finding, and it will be exciting to explore the potential implications of *IRX1* in RPh. Our focus in this study was on understanding the genetic underpinnings of vasoconstriction, but we recognize the broader physiological processes that may also be involved in the condition, including those related to thermoregulation and metabolism.

### Therapeutic implications and α2-AR antagonism

We appreciate Dr. Flavahan’s comments on the challenges associated with targeting α2-ARs for therapeutic purposes. The balance between reducing smooth muscle constriction and potentially increasing sympathetic outflow is an area of intense scientific exploration, which we plan to examine further as well. We agree that localized delivery of α2-AR antagonists could hold promise, and interventions that target cold-induced sympathetic outflow may offer additional avenues for treatment. We remain optimistic that ongoing research will help refine these therapeutic strategies, ultimately improving outcomes for patients with RPh.

The letter by Dr. Eid also states that we would claim higher *ADRA2A* expression in individuals with RPh. However, this interpretation presented in the commentary is not correct. Instead, we show that the risk allele of *ADRA2A* that associates with RPh also associates with higher *ADRA2A* expression in tibial arteries in the normal population and that this risk allele had a higher allele frequency in the colder northern parts of Finland in comparison to the southern parts of Finland.^1^

Finally, regardless of the well-described physiological contribution of α2C-ARs in human cutaneous SMC contraction upon cold exposure, large population-level human genetic studies, such as ours, showed a strong association with the α2A-AR (*ADRA2A* genetic locus) and RPh.^1^^,^^2^ This association is robust across different population cohorts.^1^ Recent data from the Million Veterans Program (MVP)^9^ (a male-biased cohort) show the same association between *ADRA2A* and RPh (PheWeb MVP: https://phenomics.va.ornl.gov/pheweb/gia/eur/pheno/Phe_443_1, accessed December 9, 2024), highlighting the significance of this finding. Interestingly, genetic association with the *ADRA2C* locus is not witnessed in any of these cohorts. Future RPh studies should, therefore, address this by taking into account the potential unique and joint signaling between the genetic risk factors led by *ADRA2A*, *ADRA2C*, and all the physiological mechanisms with α2-AR involvement upon cold exposure alongside other genetic risk factors in RPh, such as the *IRX1* locus.

In conclusion, we thank Dr. Flavahan and Dr. Eid again for their valuable feedback. We believe that constructive dialogue, such as this, is essential to advancing our understanding of complex conditions like RhP. We look forward to future opportunities for collaboration and further exploration of these important questions.

## Declaration of interests

The authors declare no competing interests.
